# Phylogenomic analysis provides diagnostic tools for the identification of *Anastrepha fraterculus* (Diptera: Tephritidae) species complex

**DOI:** 10.1111/eva.13589

**Published:** 2023-08-30

**Authors:** Carlos Congrains, Julian R. Dupuis, Erick J. Rodriguez, Allen L. Norrbom, Gary Steck, Bruce Sutton, Norma Nolazco, Reinaldo A. de Brito, Scott M. Geib

**Affiliations:** ^1^ U.S. Department of Agriculture‐Agricultural Research Service, Daniel K. Inouye U.S. Pacific Basin Agricultural Research Center, Tropical Pest Genetics and Molecular Biology Research Unit Hilo Hawaii USA; ^2^ Department of Plant and Environmental Protection Services University of Hawaii at Manoa Honolulu Hawaii USA; ^3^ Department of Entomology University of Kentucky Lexington Kentucky USA; ^4^ Division of Plant Industry, Florida Department of Agriculture and Consumer Services Gainesville Florida USA; ^5^ Systematic Entomology Lab USDA, ARS c/o Smithsonian Institution Washington DC USA; ^6^ Department of Entomology (Research Associate), National Museum of Natural History Smithsonian Institution Gainesville Florida USA; ^7^ Centro de Diagnóstico de Sanidad Vegetal, Servicio Nacional de Sanidad Agraria Peru; ^8^ Departamento de Genética e Evolução Universidade Federal de São Carlos São Carlos São Paulo Brazil

**Keywords:** insect pest, introgression, phylogenomics, species discrimination

## Abstract

Insect pests cause tremendous impact to agriculture worldwide. Species identification is crucial for implementing appropriate measures of pest control but can be challenging in closely related species. True fruit flies of the genus *Anastrepha* Schiner (Diptera: Tephritidae) include some of the most serious agricultural pests in the Americas, with the *Anastrepha fraterculus* (Wiedemann) complex being one of the most important due to its extreme polyphagy and wide distribution across most of the New World tropics and subtropics. The eight morphotypes described for this complex as well as other closely related species are classified in the *fraterculus* species group, whose evolutionary relationships are unresolved due to incomplete lineage sorting and introgression. We performed multifaceted phylogenomic approaches using thousands of genes to unravel the evolutionary relationships within the *A. fraterculus* complex to provide a baseline for molecular diagnosis of these pests. We used a methodology that accommodates variable sources of data (transcriptome, genome, and whole‐genome shotgun sequencing) and developed a tool to align and filter orthologs, generating reliable datasets for phylogenetic studies. We inferred 3031 gene trees that displayed high levels of discordance. Nevertheless, the topologies of the inferred coalescent species trees were consistent across methods and datasets, except for one lineage in the *A. fraterculus* complex. Furthermore, network analysis indicated introgression across lineages in the *fraterculus* group. We present a robust phylogeny of the group that provides insights into the intricate patterns of evolution of the *A. fraterculus* complex supporting the hypothesis that this complex is an assemblage of closely related cryptic lineages that have evolved under interspecific gene flow. Despite this complex evolutionary scenario, our subsampling analysis revealed that a set of as few as 80 loci has a similar phylogenetic resolution as the genome‐scale dataset, offering a foundation to develop more efficient diagnostic tools in this species group.

## 1 INTRODUCTION

Arthropod pests are responsible for about 20% of losses of worldwide crop production, causing a major negative impact on agriculture (Culliney, 2014; Oerke et al., 1994). The true fruit flies (Tephritidae) are a very diverse group, which includes some of the most important fruit pests in the world (Norrbom, 2004; White & Elson‐Harris, 1992). These fruit flies are mostly phytophagous, and their larvae feed on a wide variety of fruits and vegetables, which can cause direct food commodity losses and reduce trade due to quarantine regulations for imported products (Aluja & Mangan, 2008). Despite their economic relevance, the taxonomy of some of these species is not fully resolved, mainly due to the great diversity of most of the pest‐containing genera, their genetically and morphologically close relationships, and the existence of species complexes such as the *Ceratitis* FARQ complex, *Bactrocera dorsalis* complex, and *Anastrepha fraterculus* complex (Hendrichs et al., 2015; Schutze et al., 2017; Zhang et al., 2021). These taxonomic issues hinder correct species identification and as a consequence, the application of appropriate pest management strategies.

Species of *Anastrepha* are widely distributed through the tropical and subtropical parts of the Americas and comprise at least 328 species, which presently are classified into 33 species groups (Aluja, 1994; Foote, 1980; Norrbom et al., 1999, 2012, 2021; Steck et al., 2019). We follow taxonomic standards and refer to species groups within *Anastrepha* without a genus abbreviation (e.g., “*striata* group”). One of the most economically important species groups is the *fraterculus* group, which comprises 34 species (not considering morphotypes of the *A. fraterculus* complex which likely comprise multiple cryptic species) (Aluja, 1994; Norrbom et al., 2012, 2021). Several species of this group, including *A. fraterculus* (Wiedemann), sensu *lato*, *Anastrepha obliqua* (Macquart), and *Anastrepha ludens* (Loew), are recognized as major pests because of their wide distribution and capacity to infest a broad variety of fleshy fruits (polyphagy) (Hernández‐Ortiz et al., 2019; Zucchi, 2000a; Zucchi & Moraes, 2008). Nevertheless, the evolutionary relationships and taxonomy of *Anastrepha* species are still not completely clarified, with much of the taxonomy being based on morphological attributes of the adult female. Morphology‐based species identification largely relies on characters of the female terminalia, though some taxonomic keys to the immature life stages of a limited number of species have been developed (Canal et al., 2015; Frías et al., 2008; Steck et al., 2019; Zucchi, 2000b). Furthermore, the existence of overlapping measurements in some of the key taxonomic traits used, such as measurements of the tip of the aculeus of the ovipositor (Perre et al., 2014), makes species identification in some instances a challenge even for taxonomic specialists. This problem may limit control of the pests, for example, where only larvae may be detected, and accurate identification is critical for regulatory action.

Despite great efforts to resolve evolutionary relationships among *Anastrepha* species, phylogenetic studies based on molecular and morphological markers used to date have shown limited results (Barr et al., 2005; McPheron et al., 1999; Mengual et al., 2017; Norrbom et al., 1999; Silva & Barr, 2008). For instance, in the most complete molecular phylogeny of *Anastrepha* based on six DNA regions (Mengual et al., 2017), only seven of 15 investigated species groups with more than one included species were corroborated as monophyletic or nearly so, and the relationships among the species groups are largely unresolved. Phylogenies among species that belong to the same group based on one or few molecular markers also show limited resolution, particularly for some species in the *fraterculus* species group (Mengual et al., 2017; Scally et al., 2016; Smith‐Caldas et al., 2001). This pattern has been explained by a combination of large population sizes, recent diversification, retained ancestral polymorphism after speciation, and introgression (Congrains et al., 2021; Díaz et al., 2018; Scally et al., 2016).

The most enigmatic biological entities of the *fraterculus* group are undoubtedly the complex of cryptic species included in *A. fraterculus* (*s.l*.) as indicated by genetic, chemical, morphological, and ecological variation among populations distributed across the Neotropics (Cáceres et al., 2009; Hernández‐Ortiz et al., 2012; Prezotto et al., 2019; Selivon et al., 2004, 2005, 2022; Smith‐Caldas et al., 2001; Stone, 1942; Vaníčková, Břízová, et al., 2015). Based on morphometric analyses of wings and female terminalia, eight morphotypes have been recognized within the *fraterculus* complex across its distribution: Mexican (Mexico, Panama and Guatemala), Venezuelan (Caribbean lowlands of Venezuela), Andean (highlands of Venezuela and Colombia), Peruvian (lowlands of Ecuador and Peru), Ecuadorian (highlands of Ecuador and Peru), Brazilian‐1 (Brazil, Argentina, highland Peru), Brazilian‐2 (southeastern of Brazil), and Brazilian‐3 (southeastern of Brazil) (Hernández‐Ortiz et al., 2012, 2015). Analysis of the full length (~550 bp) nuclear ribosomal internal transcribed spacer 1 (ITS1) has revealed that ITS1 types (haplotypes) are associated to six morphotypes (Prezotto et al., 2019; Sutton et al., 2015). This molecular approach has also indicated broader distributions of the Mexican (Costa Rica, Colombia, and Venezuela), Peruvian (Colombia and inter‐Andean valleys of Peru), and Brazlian‐1 (Paraguay and Bolivia) morphotypes. Nevertheless, robust characterization and support for phylogenetic relationships among different *A. fraterculus* morphotypes using comprehensive sampling across its geographic range and a broad set of loci have been limited (Congrains et al., 2021; Mengual et al., 2017; Scally et al., 2016; Smith‐Caldas et al., 2001).

The complex evolutionary history of the genus *Anastrepha*, and of the *fraterculus* group in particular, limits the application of traditional barcoding or single gene approaches for species discrimination. Traditional DNA barcoding uses a region of the mitochondrial gene cytochrome oxidase I (COI) for molecular identification of animal species (Hebert et al., 2003). An evaluation of nearly 600 bp of COI revealed that approximately 75% of *Anastrepha* species can successfully be identified using this approach, but it does not discriminate members of the *A. fraterculus* cryptic complex and some other closely related species of the *fraterculus* group (Barr et al., 2018; Bartolini et al., 2020). On the other hand, genomic data coupled with multispecies coalescent approaches have provided invaluable information on evolutionary histories of cryptic species complexes in a great variety of animal taxa, such as insects, mammals, reptiles, and birds (Dupuis, Bremer, et al., 2018; Herrera et al., 2022; Obiol et al., 2023; Singhal et al., 2018; Thawornwattana et al., 2018; Zhang et al., 2021), as well as plants, such as ferns and angiosperms (Kinosian et al., 2020; Wu et al., 2018).

Disentangling the evolutionary relationships of the *fraterculus* species group is essential to establish a baseline for molecular species identification, which is critical for applying appropriate pest biocontrol measures. For example, the sterile insect technique (SIT) is a powerful and relatively environment‐friendly approach, which consists of flooding a region with sterile male insects that will mate with wild females and suppress the population. This method is highly impactful when the released males are of the same species/strain of the pest population, but would be completely ineffective if the sterile and target populations are not conspecific or if their mating behavior is incompatible, making identification critical for its success (Klassen & Vreysen, 2021; Nagel & Peveling, 2021). Despite strong genetic evidence for historical gene flow among species in the *fraterculus* group, introgression has not completely blurred species identities, so much so that laboratory experiments have shown that individuals prefer to cross with individuals of the same species and same *A. fraterculus* morphotypes (Devescovi et al., 2014; Rull et al., 2013; Santos et al., 2001). In this scenario, the identification of evolutionary lineages that share ecological and physiological attributes is essential to help establish species‐specific markers that can be useful for many different approaches, such as SIT.

Here, we used genomic information to seek a panel of phylogenetically informative loci that may be useful for the discrimination of species in the *fraterculus* species group. Our analysis was based on a phylogenomic framework focusing on unraveling the evolutionary relationships among members of the *A. fraterculus* species complex and closely related species. The first goal of this study was to develop a workflow to infer and filter orthologs to reconstruct robust species trees of this group based on diverse phylogenetic approaches (multispecies coalescent methods and concatenation) and high throughput sequencing data (transcriptome assemblies, whole‐genome assemblies, and whole‐genome resequencing). While genome‐scale phylogenies provide high resolution, the application of whole‐genome surveys in diagnostic protocols is impractical due to time, cost, and resources. Using the robust phylogeny generated, we were able to test whether a previously proposed set of 123 nuclear genes (Congrains et al., 2021) retains enough phylogenetic information to solve even the intricate relationships of *A. fraterculus* complex lineages. Furthermore, we performed a subsampling analysis of these informative genes to determine the minimum number of loci that may be used for molecular identification purposes of the species and populations sampled.

## 2 METHODS

### 2.1 Sampling and data collection

This dataset includes newly generated whole‐genome sequences for 13 specimens collected from South America and Mexico with an emphasis on *Anastrepha* taxa which are not represented in other datasets, especially lineages of the *A. fraterculus* complex (Table 1). Most of these were morphologically identified by Norrbom and Rodriguez using the key of Norrbom et al. (2012). We also included transcriptomes and genomes from previously published sources totaling 36 samples of *Anastrepha* spp. and five genomes of other genera of Tephritidae (Table 1). A full taxa list including geographic location, collection date, sex, life developmental stage, type of genomic‐scale data and corresponding reference when appropriate is provided in Table 1. Notably, the *Anastrepha* sampling included 24 specimens of species of the *fraterculus* group, of which 17 belong to the *A. fraterculus* complex.

**TABLE 1:** Sampling information.

Sample	Unique identifier	Species group	Geographic location^b^	Altitude	Coordinates	Collection Date^d^	Collector	Sex^e^	Life Stage	Type of data	Source^g^
*A. fraterculus* SP1^a^	–	*fraterculus*	Brazil: SP, São Carlos	810 m^c^	22°1′49.84″ S	–	–	F	Adult	Transcriptome	Díaz et al. (2021)
					47°54′27.90″ W						
*A. fraterculus* ES	SAMN17691821	*fraterculus*	Brazil: ES, Muniz Freire	580 m^c^	20°27′52.13″ S	X‐2015	I. Pinto	F	Adult	Transcriptome	Congrains et al. (2021)
					41°24′54.88″ W						
*A. fraterculus* RJ	SAMN17691822	*fraterculus*	Brazil: RJ, Conceição	5 m^c^	23°1′54.32″ S	II‐2015	–	F	Adult	Transcriptome	Congrains et al. (2021)
			do Jacarei		44° 9′54.14″ W						
*A. fraterculus* RS1	SAMN17691823	*fraterculus*	Brazil: RS, Dois Irmãos	10 m^c^	29°57′7″ S	04‐XII‐2016	M. J. Muller	F	Adult	Transcriptome	Congrains et al. (2021)
					51°11′33″ W						
*A. fraterculus* AR	SAMN05554138	*fraterculus*	Argentina: TUC,	340 m^c^	–	2015	‐	F	Adult	Transcriptome	SRR4026776
			Tucumán								
*A. fraterculus* SC	SAMN17691824	*fraterculus*	Brazil: SC, Itapema	0 m^c^	27°05′36″ S	27‐I‐2016	R. A. Brito	F	Adult	Transcriptome	Congrains et al. (2021)
					48°37′08″ W						
*A. fraterculus* SP2	SAMN17691825	*fraterculus*	Brazil: SP, Porto Ferreira	570 m^c^	21°50′59″ S	VIII‐2015	R. A. Brito	F	Adult	Transcriptome	Congrains et al. (2021)
					47°29′42″ W						
*A. fraterculus* BA	SAMN17691826	*fraterculus*	Brazil: BA, Ubaitaba	60 m^c^	14°18′37.66″ S	13‐X‐2016	E. A. Miranda	F	Adult	Transcriptome	Congrains et al. (2021)
					39°19′18.08″ W						
*A. fraterculus* SP3	SAMN17691827	*fraterculus*	Brazil: SP, Ilha Bela	30 m^c^	23°47′19.52″ S	18‐I‐2016	L. Zuffo	F	Adult	Transcriptome	Congrains et al. (2021)
					45°21′42.02″ W						
*A. fraterculus* RS2^a^	AP20160307.44	*fraterculus*	Brazil: RS, Vacaria	–	–	II‐2016	–	M	Adult	WGRS^f^	This study
*A. fraterculus* MX^a^	AP20160307.51	*fraterculus*	Mexico: CDMX	–	–	II‐2016	–	F	Adult	WGRS^f^	This study
*A. fraterculus* CUS	SAMN31976350	*fraterculus*	Peru: CUS, Calca	2934 m	13°19′18.18″ S	18‐III‐2014	J. Alvarez &	–	–	WGRS^f^	This study
					71°57′24.9″ W		A. Alfaro				
*A. fraterculus* LOR	AP20160316.02	*fraterculus*	Peru: LOR, Iquitos	111 m	03°15′25.27″ S	2‐II‐2015	E. J. Rodriguez &	F	Adult	WGRS^f^	This study
					72°54′28.44″ W		J. Caballero				
*A. fraterculus* ANC	AP20160316.32	*fraterculus*	Peru: ANC, Casma	40 m^c^	09°29′07.42″ S	23‐VIII‐2006	N. Nolazco	F	Adult	WGRS^f^	This study
					78°17′58.52″ W						
*A. fraterculus* MDD	AP20160315.25	*fraterculus*	Peru: MDD, Puerto	280 m^c^	12°33′28.33″ S	14‐IV‐2014	T. Perez	–	Larva	WGRS^f^	This study
			Maldonado		70°06′31.36″ W						
*A. fraterculus* CO	AP20160318.11	*fraterculus*	Colombia: RIS, Apía	1413 m	05°11′18.85″ N	12‐VIII‐2014	D. Garcia	F	Adult	WGRS^f^	This study
					75°52′28.42″ W						
*A. fraterculus* EC	AP20160307.04	*fraterculus*	Ecuador: P,	2,530 m^c^	00°12′02.70″ S	01‐IV‐2015	M. Aguilar &	F	Adult	WGRS^f^	This study
			Guayllabamba		78°28′19.99″ W		P. Ponce				
*A. turpiniae* MG	SAMN17691828	*fraterculus*	Brazil: MG, Três Marias	540 m^c^	18°12′13.28″ S	01‐III‐2015	R. A. Brito	F	Adult	Transcriptome	Congrains et al. (2021)
					45°14′23.34″ W						
*A. turpiniae* SP	SAMN17691829	*fraterculus*	Brazil: SP, Araraquara	640 m^c^	21°48′55.81″ S	26‐I‐2016	F. Torres &	F	Adult	Transcriptome	Congrains et al. (2021)
					48°12′5.34″ W		C. Congrains				
*A. distincta* SP	SAMN17691830	*fraterculus*	Brazil: SP, São Carlos	810 m^c^	21°57′33″ S	16‐I‐2016	R. A. Brito &	F	Adult	Transcriptome	Congrains et al. (2021)
					47°53′54″ W		C. Congrains				
*A. obliqua* RJ	SAMN17691831	*fraterculus*	Brazil: RJ, Conceição	5 m^c^	23°1′54.32″ S	II‐2015	‐	F	Adult	Transcriptome	Congrains et al. (2021)
			do Jacarei		44°9′54.14″ W						
*A. obliqua* GO^a^	–	*fraterculus*	Brazil: GO, Goiânia	780 m^c^	16°41′58″ S	–	–	F	Adult	Transcriptome	Díaz et al. (2021)
					49°16′35″ W						
*A. obliqua* PR1	SAMN17691832	*fraterculus*	Brazil: PR, Capanema	370 m	25°39′45.54″ S	II‐2015	F. Torres &	F	Adult	Transcriptome	Congrains et al. (2021)
					53°48′28.74″ W		R. A. Brito				
*A. obliqua* PR2	SAMN17691833	*fraterculus*	Brazil: PR, Marialva	540 m^c^	23°30′56.68″ S	II‐2015	C. Congrains &	F	Adult	Transcriptome	Congrains et al. (2021)
					51°49′34.11″ W		R. A. Brito				
*A. obliqua* CO^a^	–	*fraterculus*	‐	–	–	III‐2020	–	M	Adult	Genome	GCA_027943255.1
*A. suspensa* US^a^	–	*fraterculus*	–	–	–	2015	–	–	Adult	Genome	Dupuis, Bremer, et al. (2018)
*A. ludens* US^a^	–	*fraterculus*	–	–	–	03‐IV‐2018	–	F	Adult	Genome	GCA_028408465.1
*A. psidivora*	SAMN31976356	*insertae sedis*	Peru: CUS, Villa Carmen	534 m	12°53′42″ S	4‐III‐2013	E. Rodriguez	–	–	WGRS^f^	This study
			Research Station		71°24′10″ W						
*A. bistrigata*	SAMN17691834	*striata*	Brazil: SP, São Carlos	810 m^c^	22°1′49.84″ S	01‐III‐2015	–	F	Adult	Transcriptome	Congrains et al., 2021)
					47°54′27.90″ W						
*A. striata*	SAMN31976357	*striata*	Peru: CUS, Villa Carmen	534 m	12°53′42.23″ S	2‐III‐2013	E. Rodriguez	–	–	WGRS^f^	This study
			Research Station		71°24′09.98″ W						
*A. pseudoparallela*	SAMN17691835	*pseudoparallela*	Brazil: SP, Porto Ferreira	570 m^c^	21°50′59″ S	VIII‐2015	R. A. Brito	F	Adult	Transcriptome	Congrains et al. (2021)
					47°29′42″ W						
*A. grandis*	SAMN17691836	*grandis*	Brazil: SP, Porto Ferreira	570 m^c^	21°50′59″ S	01‐II‐2016	R. A. Brito	F	Adult	Transcriptome	Congrains et al. (2021)
					47°29′42″ W						
*A. serpentina*	SAMN17691837	*serpentina*	Brazil: SP, Araraquara	640 m^c^	21°48′55.81″ S	06‐VII‐2015	F. Torres &	F	Adult	Transcriptome	Congrains et al. (2021)
					48°12′5.34″ W		C. Congrains				
*A. hadracantha*	SAMN31976358	*mucronata*	Peru: MDD, Los Amigos	284 m	12°33′11.23″ S	13‐XII‐2013	J. Caballero &	–	–	WGRS^f^	This study
			Biological Station		70°06′38.27″ W		T. Perez				
*A. leptozona*	SAMN31976359	*leptozona*	Peru: CUS, Villa Carmen	721 m	12°53′42″ S	14‐II‐2013 to	E. J.Rodriguez	–	–	WGRS^f^	This study
			Research Station		71°24′10″ W	21‐II‐2013					
*A. curitis*	SAMN31976360	*leptozona*	Peru: MDD, Los Amigos	254 m	12°34′00.98″ W	05‐XII‐2013	J. Caballero	–	–	WGRS^f^	This study
			Biological Station		70°06′05.72″ S						
*R. zephyria*	–	–	–	–	–	–	–	–	–	Genome	GCF_001687245.1
*C. capitata*	–	–	–	–	–	–	–	–	–	Genome	Papanicolaou et al. (2016)
*Z. cucurbitae*	–	–	–	–	–	–	–	–	–	Genome	Sim & Geib (2017)
*B. dorsalis*	–	–	–	–	–	–	–	–	–	Genome	GCF_000789215.1
*B. oleae*	–	–	–	–	–	–	–	–	–	Genome	GCF_001188975.1

^a^ Samples collected from colonies.

^b^ Geographic information is shown in the format: Country: Abbreviation of State/Department/Province, City/Locality. Full names are SP: São Paulo, ES: Espíritu Santo, RJ: Rio de Janeiro, RS: Rio Grande do Sul, SC: Santa Catarina, BA: Bahia, GO: Goiás, PR: Paraná, MG: Minas Gerais, TUC: Tucumán, CDMX: Ciudad de México, CUS: Cusco, LOR: Loreto, ANC: Ancash, MDD: Madre de Dios, RIS: Risaralda, and P: Pichincha.

^c^ These altitudes and coordinates were obtained using Google Earth Pro v. 7.3.4.

^d^ Collection date are formatted as day ‐ month in Roman numerals ‐ year.

^e^ M: Male and F: Female.

^f^ WGRS: Whole‐genome resequencing.

^g^ Reference or accession number in the NCBI.

### 2.2 Laboratory procedures

To prepare and sequence whole‐genome DNA libraries of the 13 new *Anastrepha* specimens, we performed DNA extractions following the protocol in Dupuis, Sim, et al. (2018). We homogenized the tissue of the entire flies using FastPrep 24 homogenizer (MP Biomedical). Homogenized tissue was digested with proteinase K (Omega, BioTek) for 3–12 h at 55°C. We then extracted genomic DNA using the Mag‐Bind Tissue DNA KF Kits (Omega, BioTek) in an automatic extractor instrument (KingFisher Flex‐96, Fisher Thermo Scientific) following manufacturer's instructions with RNase A treatment. Whole‐genome resequencing (WGRS) DNA libraries (Table 1) were prepared using NEBNext Ultra II DNA Library Prep Kits for Illumina (New England BioLabs) following the manufacturer's instructions. Libraries were quantified using a Fragment Analyzer Automated Capillary Electrophoresis System with HS Genomic DNA Kit (Advanced Analytical Technology), and barcoded to be pooled into two final libraries containing six and seven individuals, respectively, which were each sequenced with pair‐end (PE) sequencing (2 × 150 bp) in an individual lane of the Illumina HiSeq X platform (Beijing Genomics Institute).

### 2.3 Quality filtering

The shotgun sequence data for the 13 samples were initially inspected using FastQC software (https://www.bioinformatics.babraham.ac.uk/projects/fastqc/). Illumina adapters and low‐quality sequences were removed using the Trimmomatic v. 0.39 program (Bolger et al., 2014). We set HEADCROP = 1, LEADING = 5, TRAILING = 5, SLIDINGWINDOW:5:20, MINLEN = 50 and default parameters for removing the adapters in Trimmomatic v. 0.39.

### 2.4 Coding sequences prediction

We predicted putative coding sequences (CDSs) of publicly available assembled genomes of *A. obliqua* (GCA_027943255.1), *A. ludens* (GCA_028408465.1), and *Anastrepha suspensa* (Loew) (Dupuis, Bremer, et al., 2018) using Augustus v. 3.4 (Stanke et al., 2006) and Scipio v. 1.4.1 (Keller et al., 2008). We performed an ab initio gene prediction in the program Augustus using the training set for *Drosophila melanogaster* Meigen. We added protein information by aligning the set of proteins predicted for the *Anastrepha bistrigata* Bezzi transcriptome to each genome using BLAT tool (Kent, 2002) as implemented in Scipio. The other parameters of Scipio were set as default. The results from both approaches were joined and the redundant putative CDSs were reduced using a sequence identity threshold of 0.99 and the other parameters as default in CD‐HIT‐est (Fu et al., 2012; Li & Godzik, 2006).

### 2.5 Ortholog inference

Clusters of orthologs were inferred from CDSs of 20 transcriptomes of *Anastrepha* and genomes of five other tephritids, including *Rhagoletis zephyria* Snow, *Ceratitis capitata* (Wiedemann), *Zeugodacus cucurbitae* (Coquillett), *Bactrocera oleae* (Rossi), and *Bactrocera dorsalis* (Hendel) (see Table 1 for details), applying a phylogenetic‐based approach (Yang & Smith, 2014), then filtered using POTION pipeline (Hongo et al., 2015), performing the procedures described in Congrains et al. (2021). We conducted the procedures of aTRAM v. 2.3.3 pipeline (Allen et al., 2018) to predict the same set of orthologs for the 13 WGRS of *Anastrepha* using trimmed reads from each sample as databases and consensus sequences of ortholog clusters as the query for BLAST searches (Camacho et al., 2009). We used default parameters in aTRAM, except that we set Trinity v. 2.5.1 (Grabherr et al., 2011) as the assembler and a word size of four.

We also incorporated protein predictions from genome assemblies of *A. obliqua*, *A. ludens*, and *A. suspensa* using BLASTn searches (Camacho et al., 2009), where the predicted CDSs were used as databases and consensus sequences of the ortholog clusters as queries. As we expected these orthologs to be single copy in the genomes based on the orthology analysis, the alignment with the best score of each comparison was retained and treated as the corresponding ortholog.

**FIGURE 1:**
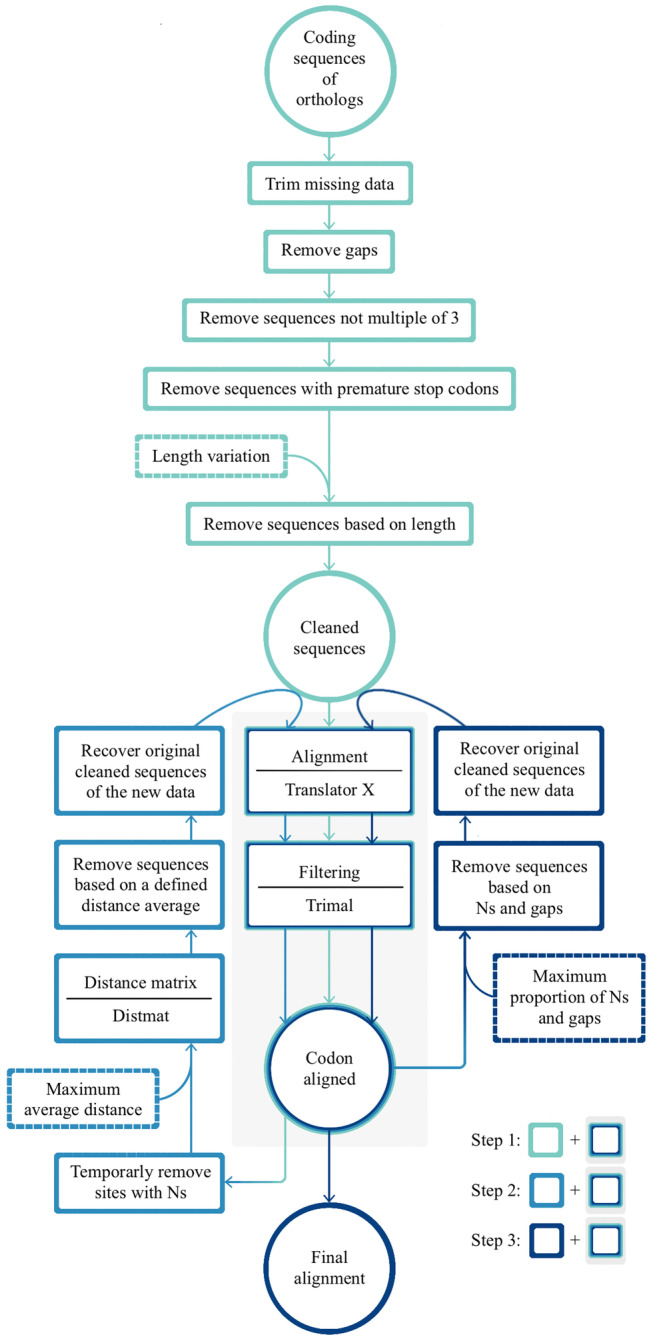
The phylosweeper framework. This python script performs three filtering steps, which are controlled by three parameters that are indicated by the user (dashed‐line boxes). In the first step, the user defines a length variation cutoff. For example, a 5% cutoff means that sequences with a length greater than 105% or lower than 95% of the average length will be removed. In the second step, the user defines the maximum average of uncorrected pairwise distance. Possible values range from 0 to 100. In the third step, the user provides the maximum fraction of allowed missing data or gaps. For example, 0.05 means that sequences with more than 5% of missing data or gaps will be excluded. In steps 2 and 3, the alignments are performed using the trimmed sequences produced by step 1 but include only those that passed the filter.

The set of orthologs was divided into two datasets: one included all samples that we refer to as the *Anastrepha* set, whereas the second, named the *fraterculus* group set, included all samples from the *fraterculus* group (*A. fraterculus* complex and related species), the *incertae sedis Anastrepha psidivora* Norrbom, and two outgroups from the *striata* species group, *Anastrepha striata* Schiner and *Anastrepha bistrigata* Bezzi. For both datasets, ortholog clusters were filtered and aligned using a pipeline developed de novo as part of this study, phylosweeper (https://github.com/popphylotools/Phylosweeper.git). This python script aimed to remove potential paralogs, spurious inferred orthologs, misassembled sequences, and poorly aligned regions. It performed three steps of filtering followed by a round of alignment and trimming (Figure 1): (i) the first step was to trim missing data and remove sequence gaps. Sequences with a premature stop codon, with lengths not a multiple of three, and sets with high length variation were removed. Based on preliminary analysis of this data, we set a 30% length variation cutoff for both datasets. (ii) The second step aimed to remove distantly related sequences. For that, average distance per sample was estimated based on an un‐corrected pairwise distance matrix calculated in the DISTMAT tool of the EMBOSS package (Rice et al., 2000). A predefined average threshold was used for filtering purposes. For this study, we calculated the average, standard deviation (SD), and median of the highest genetic distance for each cluster of orthologs. For the *Anastrepha* dataset, the average was 32.75, the standard deviation was 18.58 and the median was 18.81. The high SD (more than half of the average) indicated a great variability of this parameter among the clusters, so we used the median as a reference. As the goal was to remove possible misassembled or misassigned loci to their respective cluster of orthologs, but preserve most of the genes, including the highly variable ones, we set this parameter to 25%. For the *fraterculus* group dataset, we followed the same strategy and based on an average of 17.72, a standard deviation of 20.73 and a median of 2.93, we set this parameter to 10%. iii) The third step was to avoid sequences with high missingness measured in terms of proportion of missing data (Ns) and gaps. We set a threshold of 25% missing data in both datasets. Each step was followed by a round of alignment and cleaning. The alignment consisted of translating the CDS to protein sequences and aligning these sequences using the muscle algorithm (Edgar, 2004) as implemented in the TranslatorX program (Abascal et al., 2010). The cleaning consisted of removing poorly aligned regions from protein alignments using the strictplus option in Trimal (Capella‐Gutiérrez et al., 2009). The final DNA alignment was produced by a combination of the TranslatorX codon alignment and the filtered protein alignment.

### 2.6 Phylogenetic analysis

Filtered ortholog gene alignments of the *Anastrepha* and *fraterculus* group datasets were independently used to infer gene trees. The best‐fit nucleotide substitution models of the alignments were selected based on the Bayesian information criterion (BIC) using Model‐Test‐NG (Darriba et al., 2020). In this analysis, we restricted the likelihood comparisons to the models allowed in the RAxML‐NG program (Kozlov et al., 2019). The maximum likelihood (ML) gene trees were inferred in RAxML‐NG (Kozlov et al., 2019) using the corresponding best‐fit model and 200 rapid bootstrap replicates.

The species trees were estimated by combining gene alignments into a super‐matrix using a concatenation method and by combining gene trees using multispecies coalescent approaches for both datasets. Concatenation analysis was conducted including the best‐fit model for each gene and 200 bootstrap using IQ‐TREE v. 2.1.2 (Minh, Schmidt, et al., 2020). The multi‐species coalescent trees were estimated based on ML gene trees using default parameters in ASTRAL v. 5.7.7 (Zhang et al., 2018). Phylogenetic supports were assessed by the gene concordance factor, local posterior probabilities (PP), and the quartet support. The former is the proportion of gene trees that agrees with a branch in the species tree (Minh, Hahn, & Lanfear, 2020), which was calculated by IQ‐TREE v. 2.1.2 (Minh, Schmidt, et al., 2020). Local PP and quartet support are calculated based on the frequency of quartets in the gene trees (Sayyari & Mirarab, 2016), which was estimated using ASTRAL v. 5.7.7 (Zhang et al., 2018).

### 2.7 Testing for reticulated history of the *fraterculus* species group

We inferred phylogenetic networks, which incorporate the possibility of introgression and hybridization between lineages and represent these events as hybrid edges or reticulations in phylogenetic trees (Huson & Bryant, 2006). The networks were estimated based on 3031 ortholog groups of the *fraterculus* group set (excluding the outgroups *A. psidivora*, *A. striata*, and *A. bistrigata*). We performed six inferences varying the maximum number of reticulations from 0 to 5, each estimated from 500 bootstrap replicates, and the samples were grouped in accordance with the clades in the species tree. These inferences were carried out using the Maximum Pseudolikelihood approach in Phylonet v. 3.8.2 (Than et al., 2008; Wen et al., 2018). The phylogenetic networks were visualized in PhyloNetworks (Solís‐Lemus et al., 2017).

### 2.8 Molecular discrimination of *fraterculus* group species

We inferred phylogenetic relationships using a reduced set of 129 loci that demonstrated high levels of phylogenetic resolution in terms of percentage of resolved clades (87.5% and 100%) and resistance to gene flow (Congrains et al., 2021). After excluding gene trees encompassed by fewer than 20 specimens (due to missing or filtered data), this “phylogenetically informative set” of 123 remaining trees were combined to infer a species tree, which was used to assess phylogenetic support employing ASTRAL v. 5.7.7 (Zhang et al., 2018) and IQ‐TREE v. 2.1.2 (Minh, Schmidt, et al., 2020), as described above.

The species tree produced from the complete *fraterculus* group dataset was compared to alternative sets of randomly sampled gene trees. For these comparisons, we generated 500 random sets of 10, 20, 30, 40, 50, 60, 70, 80, 90, 100, or 110 gene trees each subsampled from the informative loci (123 gene trees) and the whole dataset (3031 gene trees) generated for the *fraterculus* group set, with replacement. In the same way, we also generated subsamples of 200, 300, 400, 500, 600, 700, 800, 900, and 1000 gene trees from the whole dataset. To assure a minimum level of consistency, all subsets had phylogenetic information for each specimen in at least two gene trees. The species tree for each subsample set was estimated by ASTRAL v. 5.7.7 (Zhang et al., 2018). Furthermore, we compared the topology of the rooted species tree (using *A. bistrigata* as an outgroup) estimated from the *fraterculus* group dataset and each subset's species tree by calculating Robinson–Foulds distance (Robinson & Foulds, 1981). We also checked for the presence of the clades defining species or lineages and their relationships (grouping pattern of 17 clades of the tree) in the subsampled species trees. We performed this evaluation to determine the minimum number of genes that consistently resolve the phylogeny in the same pattern as the full dataset. This subsampling analysis and the tree comparisons were carried out using a custom python script (https://github.com/popphylotools/sampling_random_trees), which employs tools implemented in Environment for Tree Exploration (ETE) v. 3. (Huerta‐Cepas et al., 2016).

### 2.9 Location of the 123 informative loci in the genome

We selected one sequence from each cluster of orthologs of the *fraterculus* group dataset to establish the location of each marker in the *A. ludens* genome. For this analysis, we used sequences of *A. ludens*, when available, otherwise we used sequences of *A. distincta* Greene. These sequences were aligned to the *A. ludens* genome using default parameters except that the model was set to est2genome in exonerate v. 2.4.0 (Slater & Birney, 2005). We calculated pairwise distance in base‐pairs (bp), using the first coordinate estimated by exonerate and a custom python script. The visualization was performed in the R package chromoMap v0.4.1 (Anand & Rodriguez Lopez, 2022).

## 3 RESULTS

We sequenced complete genomes of 13 *Anastrepha* specimens, which included four species groups and eight samples from the *A. fraterculus* complex across South America and Mexico. The sequenced samples had between 39 and 91 million PE reads, which were approximately 15 to 35× genome coverage (using a genome size estimate of 825 Mb from the *A. ludens* genome assembly). The filtering step excluded between 5% and 7% of the raw reads (Table S1). These data were combined with previously published transcriptomes and complete genomes assemblies, totaling 41 Tephritidae samples that were used to infer evolutionary relationships among seven *Anastrepha* species groups, with a special focus on the *fraterculus* species group.

### 3.1 Phylogenetic analysis

**FIGURE 2:**
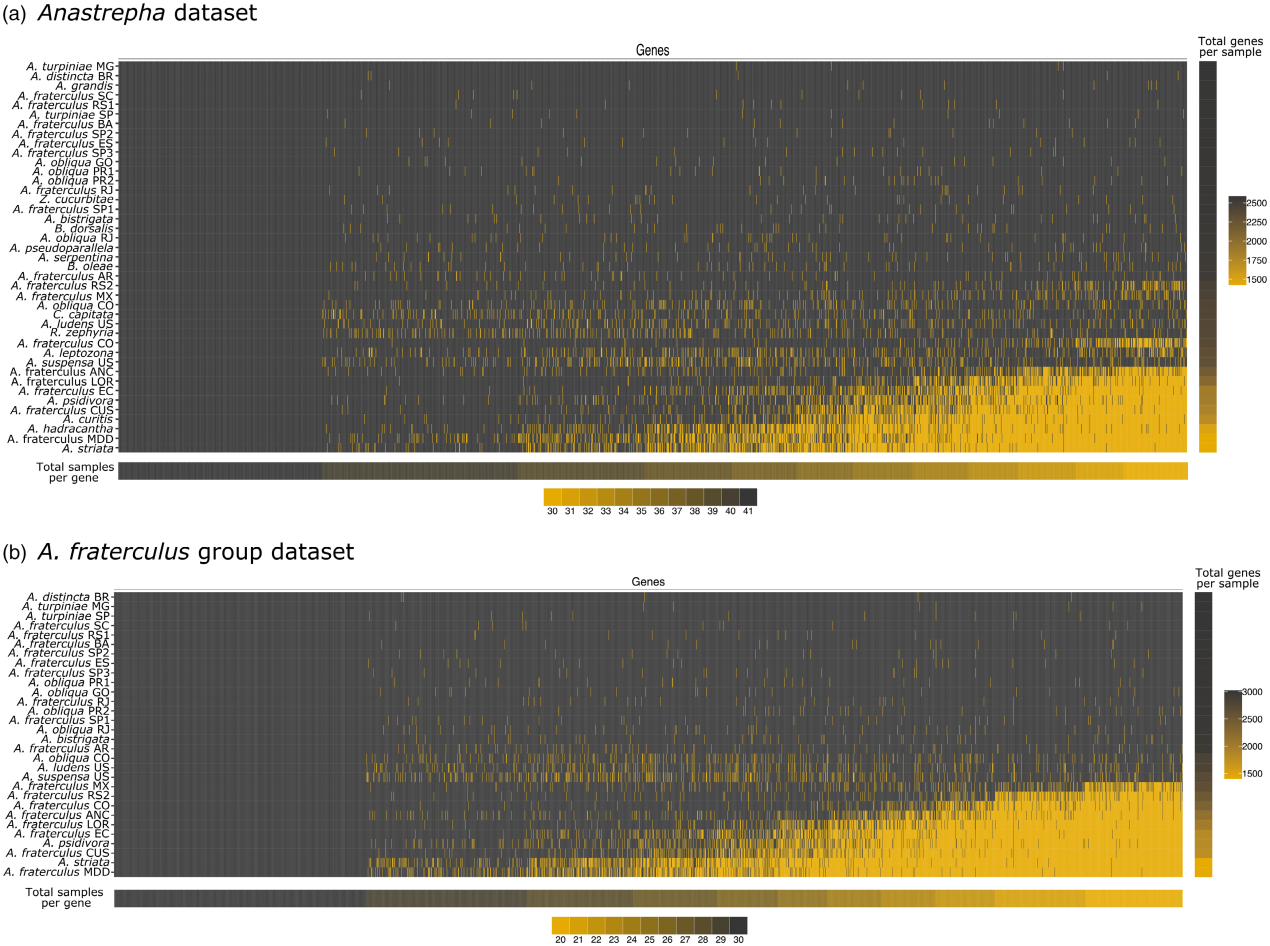
Gene occupancy matrix for *Anastrepha* and *A. fraterculus* group datasets. (a) 73% gene occupancy of *Anastrepha* dataset matrix and heatmaps of samples per gene (bottom) and ortholog genes per sample (right). (b) 67% gene occupancy of *A. fraterculus* and heatmaps of samples per gene (bottom) and number of genes per sample (right). Colors in the matrix indicate presence (black) or absence (yellow) of an ortholog gene per sample.

After the filtering and cleaning steps, we retained alignments with at least 30 (73% gene occupancy) samples for the *Anastrepha* dataset and at least 20 (67% gene occupancy) samples for the *fraterculus* group dataset (Figure 2). This procedure produced 2591 gene alignments with 2,931,129 nucleotide sites and an average length of 1131.27 bases per alignment for the *Anastrepha* dataset and 3031 gene alignments with 4,470,387 nucleotide sites and average of 1474.89 bases per alignment for the *fraterculus* group dataset.

**FIGURE 3:**
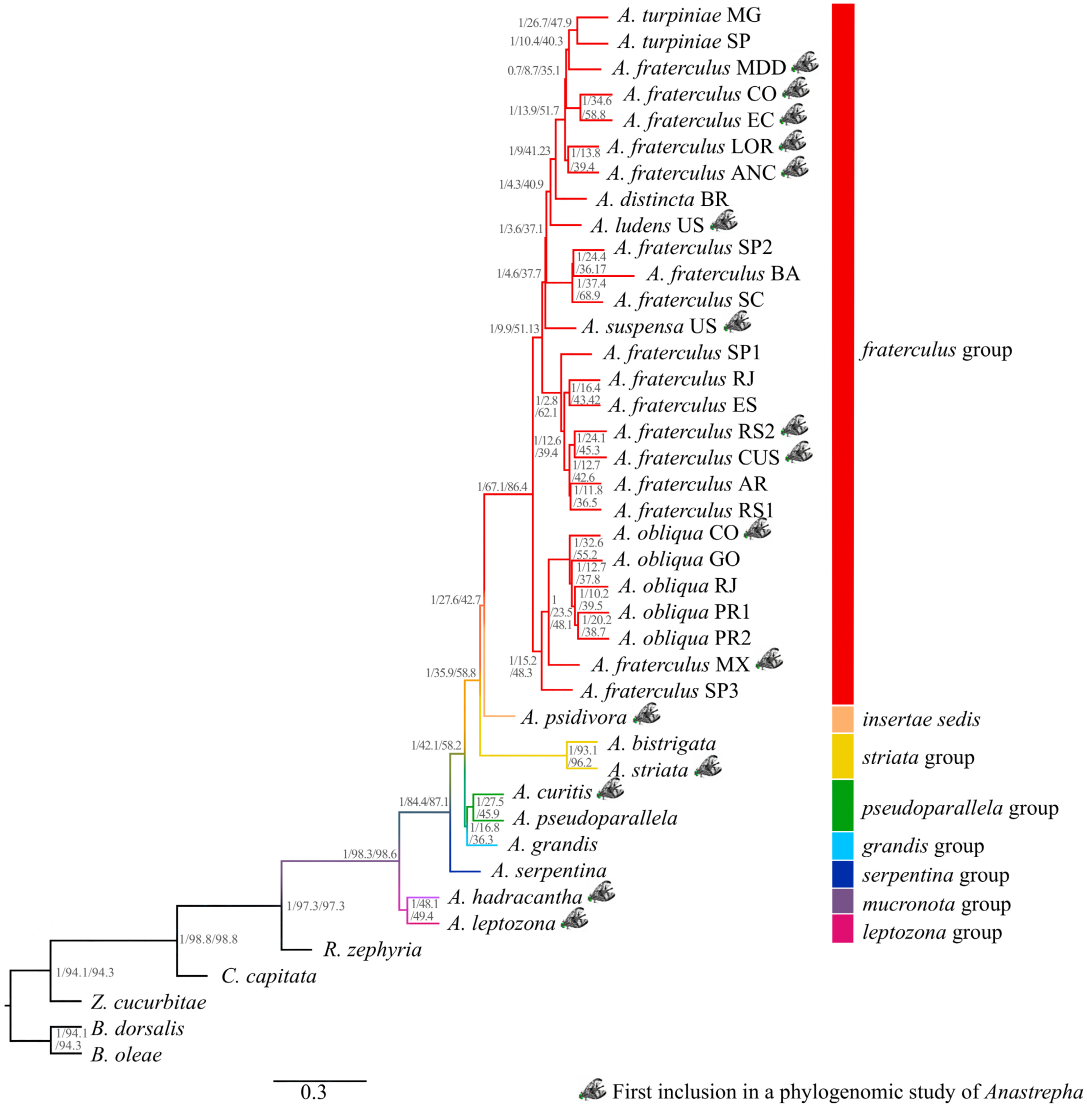
Multispecies coalescent species tree of *Anastrepha* and five other Tephritidae species as outgroups based on 2591 genes inferred in ASTRAL‐III. Phylogenetic support measured by bootstrap, gene concordant factor, and quartet support are showed close to the nodes.

**FIGURE 4:**
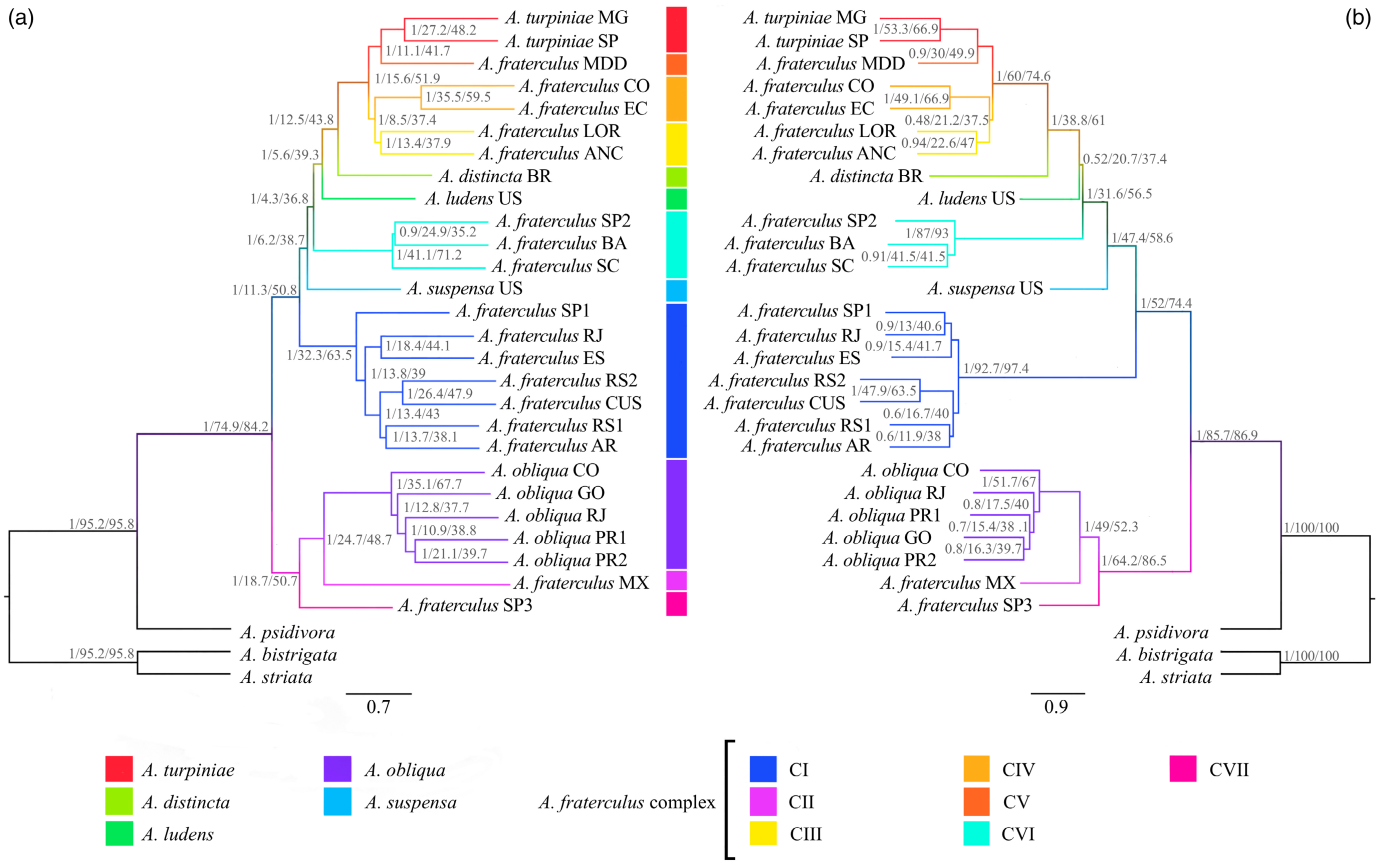
Phylogenetic analysis of the *fraterculus* group using *A. psidivora* and the *striata* group species as outgroups. Multispecies coalescent species trees were recovered based on 3031 genes (a) and 123 genes (b) in ASTRAL‐III. Phylogenetic support measured by bootstrap, gene concordant factor and quartet support are showed in that order close to the nodes.

**FIGURE 5:**
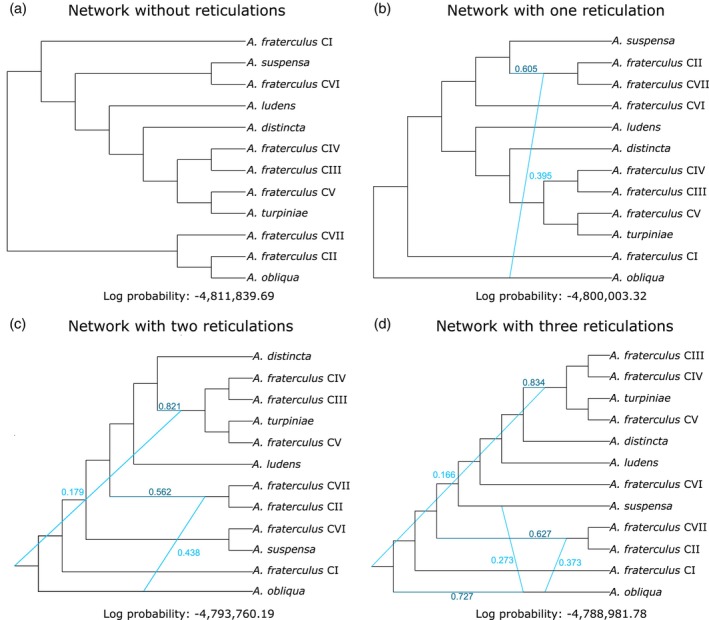
Phylogenetic networks of *fraterculus* group lineages based on 3031 gene trees inferred under maximum pseudo‐likelihood approach in Phylonet. Though we inferred networks with 0–5 reticulations allowed, only the networks with 0–3 reticulations are shown here (a–d). Inheritance probabilities (γ) are displayed in sky‐blue.

The topologies of the species trees derived from the *Anastrepha* as well as the *fraterculus* datasets using different methodological approaches were highly congruent, differing only in branching patterns involving Clade V and Clade VI of the *A. fraterculus* complex (Figures 3, 4, 5; Figures S1 and S2). We found 100% bootstrap (for the concatenated approach) and 1.0 PP (for the multispecies coalescent approach) associated with the clades of *fraterculus* group highlighted in the species trees (Figure 4; Figure S2). Despite the high support, the multispecies coalescent phylogenies for both datasets displayed a high‐level of phylogenetic incongruence across different genes, measured by relatively low values of quartet support and gene concordance factor, especially for relationships among more closely related lineages.

In the species tree for the *Anastrepha* dataset, the three species groups with more than one sample (the *fraterculus*, *striata*, and *pseudoparallela* groups) were monophyletic (Figures 3 and 4). Notably, the unplaced species, *A. psidivora*, was sister to *fraterculus* group, whereas *A*. *hadracantha* (*mucronota* group) was sister to *A. leptozona* (*leptozona* group). In addition, the lineage formed by these latter two groups was sister to the remaining *Anastrepha* groups.

A more detailed look at the *fraterculus* group showed *A. fraterculus s.l*. as a polyphyletic taxon, which may be subdivided based on branching patterns and geography into seven lineages (Figure 4a). *Anastrepha fraterculus* CI was a widely distributed group, which includes samples from the Southeast and South regions of Brazil, northwestern Argentina, and the southern Andes of Peru. *Anastrepha fraterculus* CII is formed by one specimen from Mexico, whereas *A. fraterculus* CIII encompasses one sample from the Peruvian Amazon forest and one from the Peruvian coast. *Anastrepha fraterculus* CIV is composed of two samples from the Andean region of Ecuador and Colombia, whereas *A. fraterculus* CV comprises another sample from the Peruvian Amazon. *Anastrepha fraterculus* CVI is composed of three samples, two from Southeast Brazil and one from Northeast Brazil, and *A. fraterculus* CVII is a sample from the coastal part of Southeast Brazil. *Anastrepha fraterculus* CIII, CIV, and CV formed a clade which also included *A. turpiniae* Stone, in which the members of the complex would comprise a paraphyletic group, and this lineage was sister to *A. distincta*. *Anastrepha obliqua* from Brazil and Colombia constituted a monophyletic group that is the sister group of *A. fraterculus* CII, from Mexico.

The species tree inferred by the whole dataset has the same interspecies relationships as the one produced by the phylogenetically informative set of 123 genes (Figure 4). The interspecific topology showed very high local PP, higher gene concordance factor and quartet support, except for the paraphyletic lineage that included *A. turpiniae*, *A. fraterculus* CIII, CIV, and CV, *A. distincta*, and *A. ludens* (Local PP = 0.52), indicating a higher level of phylogenetic congruence for the subsampled genes in the species tree based on the informative set.

### 3.2 Testing for reticulated history of the *fraterculus* group

Because of the high levels of phylogenetic incongruence across different species trees, we also inferred networks allowing for the possibility of reticulate history. A comparison of log pseudo‐likelihood of networks with zero to five possible reticulations established that the optimum network included three reticulations, as shown by a clear plateau between number of reticulations and log of pseudo‐likelihood (Figure S3). Networks showed high‐inheritance probability (from 0.166 to 0.438), which indicates a strong genetic contribution of the parents to the potential introgressed genome (Figure 5). Two reticulations in the optimum network involved *A. obliqua* and the reticulation edge between this lineage and the common ancestor of *A. fraterculus* CI and CVII displayed the greatest inheritance probability (Figure 5d).

### 3.3 Molecular discrimination of *fraterculus* group species

**FIGURE 6:**
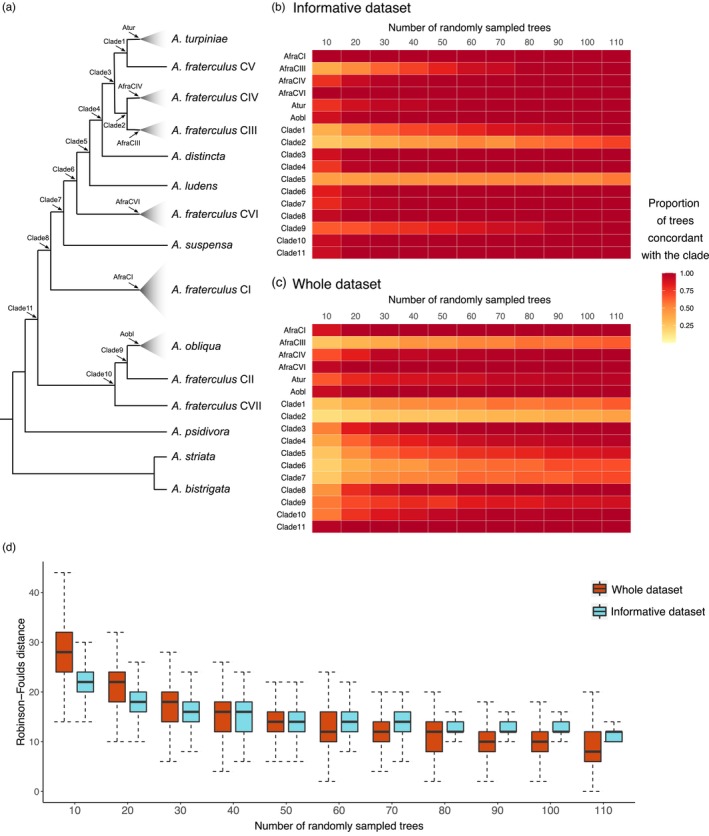
Phylogenetic congruence analysis of gene tree subsamples derived from the whole dataset and from a set of 123 highly informative loci. (a) Species tree inferred from the *fraterculus* group dataset indicating by arrows which clades were considered as surrogates for phylogenetic information. Heatmaps of the proportion of clades recovered for each class containing subsets of increasing number of gene trees (10, 20, 30, 40, 50, 60, 70, 80, 90, 100, and 110) randomly sampled from the informative genes (b) and the whole dataset (c). Each category included the information of 500 species tree inferred from independently subsampled set of gene trees. (d) Box plot showing the distribution of Robinson–Foulds distance calculated between species trees inferred from the *fraterculus* group dataset and each subset. The boxes indicate the interquartile range (IQR), the medians are shown as black lines inside the boxes and the whiskers were estimated at 1.5 IQR.

The species tree produced by the whole dataset was contrasted with those inferred from randomly sampled subsets of 500 replicates of 10, 20, 30, 40, 50, 60, 70, 80, 90, 100, and 110 independent gene trees drawn from the informative and whole dataset (Figure 6). We used the proportion of the interspecific nodes from the overall dataset, which were also recovered by the species tree of each subsample as an indication of phylogenetic resolution. Accordingly, we observed higher levels of resolution from subsamples derived from the 123 highly informative loci when compared to the subsets sampled from the whole dataset, a signal which was stronger in subsets with fewer genes (Figure 3a,c). We expected phylogenetic resolution to increase with the number of gene trees considered, until it reaches a steady‐state, which we observed in a comparison of species trees from each subset and the *fraterculus* group dataset in terms of cladogenesis and Robinson–Foulds distance (Figure 6). Subsets of 30 loci of the informative set recovered the monophyly of the *fraterculus* group lineages produced in the full dataset (i.e., *A. fraterculus* CI, CIII, CIV, and CIV, *A. turpiniae*, and *A. obliqua*) in more than 70% of the sampled species trees. Moreover, the phylogenetic signal defining those clades was stronger than the signal that supported relatively deeper clades, especially for Clade 2 and Clade 5 (Figure 6b). Notably, subsets of 80 gene trees of informative genes displayed similar phylogenetic resolution and heterogeneity among species tree topologies as categories with higher numbers of sampled trees (90, 100, and 110), which indicates that this category has reached the stationary phase (Figure 6b,d). This trend was not reached when randomly sampled loci were considered (Figure 6c). Moreover, subsets of 80 gene trees from the informative genes had similar resolution (except for Clade 5) as the subsets of 700 gene trees from the whole dataset (Figure S4).

### 3.4 Location of the 123 informative loci in the genome

**FIGURE 7:**
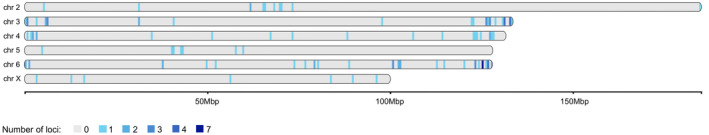
Location of the set of 123 highly informative loci in the *A. ludens* genome. The genome was divided into bins of 500 Kbp and the number of loci in each segment is indicated using different shades of blue.

The informative loci were located across the five autosomes and the X chromosome of the *A*. *ludens* genome (Figure 7). Seventy‐one loci had a distance to the closest locus higher than 100 Kbp (Figure S5). If we assume that loci located less than 100 Kpb to the closest locus are in linkage disequilibrium, there would be 97 potentially independent loci.

## 4 DISCUSSION

We conservatively inferred orthologs, developed a tool to filter out potentially misassigned orthologs and misaligned genes, and use orthologous regions across the genome to reconstruct phylogenetic relationships using transcriptomic and genomic data. Our methodology allowed us to infer the phylogeny of 41 tephritid specimens belonging to five genera and seven *Anastrepha* species groups based on more than 2000 loci. In general, phylogenetic inferences agree with the current taxonomy of these taxa (Norrbom et al., 1999; Zucchi, 2000b), as species groups are recovered as monophyletic, and samples from the same species are allocated to the same lineages, except for the *A. fraterculus* complex, which was confirmed to be polyphyletic (Congrains et al., 2021; Mengual et al., 2017; Scally et al., 2016; Smith‐Caldas et al., 2001). Furthermore, our results corroborate that the *A. fraterculus* complex, *A. obliqua*, *A. turpiniae*, and *A. suspensa* have diverged under the presence of gene flow, which has previously been suggested for those and other species of the *fraterculus* group (Congrains et al., 2021; Díaz et al., 2018; Scally et al., 2016). Despite this complex evolutionary scenario, we showed that sampling as few as 30 genes can provide resolution to most clades and sampling up to 80 or more genes may be required to have enough phylogenetic information to confidently reconstruct the phylogeny of this troublesome group. This opens the door for lower cost applied phylogenomic approaches using a multi‐gene panel to rapidly identify species when critical identifications, often on immature specimens, are needed, as in cases of exotic detection, quarantine, or importation of commodities in global trade.

Although the investigation of the whole dataset and the informative set of genes produced robust phylogenetic inferences for the genus and adequately separated lineages, we still found high levels of incongruence across different gene trees, which can be attributed to incomplete lineage sorting (ILS) and introgression (Degnan & Rosenberg, 2009). In fact, both factors have been previously reported to be main sources of gene tree discordance in *Anastrepha* (Congrains et al., 2021; Díaz et al., 2018). Because of the conflict across gene trees, branch supports should be carefully interpreted. For instance, we found high‐bootstrap values (100% for all clades defined in this study), which can be inflated because they are directly affected by the high number of genes analyzed and does not consider topological variation (Minh, Hahn, & Lanfear, 2020; Salichos & Rokas, 2013). On the other hand, some clades in the species trees displayed low‐gene concordance factors, which could be a consequence of introgression, but could also be due to insufficient information in gene alignments and short branches (Minh, Hahn, & Lanfear, 2020), which is common for rapidly diverging lineages (Saitou & Nei, 1986). Nevertheless, species tree inferences were consistent across phylogenetic approaches and datasets, except for the placement of *A. fraterculus* CV. This clade was in a different position in the phylogenies estimated using coalescent or concatenated approaches. This discrepancy may be due to the higher sensitivity of the latter method to the presence of high ILS and short branches than multispecies coalescent methods (Kubatko & Degnan, 2007; Roch & Steel, 2015).

Several multispecies coalescent methods can accommodate gene tree heterogeneity by treating it as caused by ILS but may be inconsistent under the presence of gene flow between species (Solís‐Lemus et al., 2016). Introgression among *fraterculus* group lineages has been previously reported (Congrains et al., 2021; Díaz et al., 2018), but these studies lacked the wider array of lineages from the *A. fraterculus* complex that we included in this study. The phylogenetic networks that jointly considered ILS and introgression in Phylonet supported at least three reticulations in the *fraterculus* group dataset. Two reticulations involved ancestral *A. fraterculus* complex lineages, which indicates that the introgression event involved extinct common ancestors or an unsampled species (Edelman et al., 2019). The third reticulation occurred between non‐sympatric samples of *A. obliqua* and *A. suspensa*, which may indicate signals of ancestral introgression instead of ongoing gene flow. These results clearly show that *A. fraterculus* lineages have evolved under a complex scenario involving widespread interspecific gene flow that may be better elucidated if we include genomic data at a population scale of at least the most representative species of this group.

Our phylogenomic inference agrees with the previous analysis using multispecies coalescent methods (Congrains et al., 2021) and it is generally consistent with other phylogenies based on a reduced number of genetic markers (Mengual et al., 2017; Scally et al., 2016). In the *fraterculus* group, we found that *A. obliqua* based on samples from across South America appears to be monophyletic, as indicated elsewhere (Passos et al., 2018; Scally et al., 2016), whereas *A. fraterculus s.l*. appears to be polyphyletic. However, we also noticed differences in the placement of *Anastrepha* groups compared to the phylogeny inferred by Mengual et al. (2017), which can be attributed to insufficient gene or taxon sampling. For example, the *pseudoparallela* and *grandis* groups as well as the *mucronota* and *leptozona* groups were inferred as sister groups in the ASTRAL phylogeny (Figure 3), inconsistency with the previous phylogeny (Mengual et al., 2017) that can be attributed to long branch attraction caused by taxon sampling bias (Heath et al., 2008). On the other hand, another discrepancy with the phylogeny inferred by Mengual et al. (2017) was the position of the *striata* group or *A. psidivora* as sister taxon of the *fraterculus* group, which may be due to stochastic errors caused by the high level of gene tree heterogeneity. The relationships among the lineages of the *fraterculus* group remained unaltered, although the addition of four *A. fraterculus* lineages, *A. ludens*, and *A. suspensa*, which suggests that taxon sampling would have limited effect on the accuracy of this phylogeny.

We identified seven lineages in the *A. fraterculus* complex based on their position in the species tree and geography. The existence of multiple cryptic lineages within the *A. fraterculus* complex is supported by evidence from various sources, including morphometric analyses (Hernández‐Ortiz et al., 2012, 2015). More recently, there has been indication that some of that divergence is associated with variation in the ITS1 region (Prezotto et al., 2019; Sutton et al., 2015), which makes it relevant for us to try to use that information to associate the previously recognized entities within the *A. fraterculus* complex with our phylogenomic assessment. *Anastrepha fraterculus* CI is the most widely sampled lineage in this study in terms of number of samples and geographic distribution, including samples from Brazil, Argentina, and Peru. Four of these specimens (*A. fraterculus* RS1, *A. fraterculus* ES, *A. fraterculus* AR and *A. fraterculus* RJ) have ITS1 type TI, and thus probably correspond to morphotype Brazilian‐1 and *Anastrepha* sp. 1 aff. *fraterculus* (Table 2; Congrains et al., 2021; Goday et al., 2006; Hernández‐Ortiz et al., 2012; Selivon et al., 2004, 2005; Sutton et al., 2015). Two *A. fraterculus* CVI specimens were sampled from localities close to where *Anastrepha* sp. 2 aff. *fraterculus* has been reported and they shared host fruits from the same genus (*Citrus* sp. for the sample from São Paulo) (Selivon et al., 2005), but they formed a different ITS1 clade (Congrains et al., 2021). In contrast, recent studies have associated *A*. sp. 2 aff. *fraterculus* with a subtype of TI (Tic) (Prezotto et al., 2019; Selivon et al., 2022), indicating that the lineage researched here does not correspond to this member of the complex, and that a more comprehensive sampling across Brazil should help resolve these discrepancies.

**TABLE 2:** Putative cryptic species in *Anastrepha fraterculus* complex.

Reference	This study		Hernández‐Ortiz et al. (2012, 2015)	Sutton et al. (2015)	Prezotto et al. (2019)	Goday et al. (2006), Selivon et al. (2004, 2005)	Congrains et al. (2021)
Data	Genome scale	Sampling location	Morphology	ITS1	ITS1 and morphology	Cytology and morphology	Genome scale
Nomenclature	CII^a^	Mexico	Mexican	TII	TII, TIIa, TIIb		
	CIII^a^	Lowlands of	Peruvian	TIIIA	TIIIA	sp. 4^d^	
		Peru					
	CIV^a^	Highlands of	Andean	TIV	TIV/TIVa		
		Ecuador and					
		Colombia					
	CI	Brazil,	Brazilian‐1	TI and TIa	TI, TIa,	sp. 1^d^	C1
		Peru and			TIb, TIe		
		Argentina					
	CVII^a^ ^,^ ^b^ ^,^ ^c^	Brazil	Brazilian‐2 or		TIc or	sp. 2^d^ or	C3^b^ ^,^ ^c^
			Brazilian‐3		TId	sp. 3^d^	
	CVI^c^	Brazil					C2^c^
	CV	Peru					

^a^ Association with the previous studies based only on geography.

^b^ As Brazilian morphotypes are in sympatry, it is not feasible to associate *A. fraterculus* CVII to these morphotypes based on geography.

^c^ CVI and CVII have the exact same samples as C2 and C3, respectively.

^d^ sp. 1, sp. 2, sp. 3 and sp. 4 are abbreviations for *Anastrepha* sp.1 *aff*. *fraterculus*, *Anastrepha* sp.2 *aff*. *fraterculus*, *Anastrepha* sp.3 *aff*. *fraterculus* and *Anastrepha* sp.4 *aff*. *fraterculus*, respectively.

Since we lack ITS1 sequences for the other lineages, we used geographic information to roughly associate them with the previously recognized entities of the complex based on the premise that these cryptic species are not randomly distributed (Hernández‐Ortiz et al., 2012; Prezotto et al., 2019; Sutton et al., 2015). In this way, three of the lineages can putatively be associated (Table 2); *Anastrepha fraterculus* CII with the Mexican morphotype, clade CIII with the Peruvian morphotype, and clade CIV with the Andean morphotype. The correspondence between *A*. *fraterculus* CVII to the Brazilian‐2 (subtype Tid) or Brazilian‐3 (subtype Tid) morphotypes is uncertain because the geographic distribution of the Brazilian morphotypes is not clearly defined and some populations are in sympatry (Prezotto et al., 2019). *Anastrepha fraterculus* CV comes from a single sample from a tropical forest in Puerto Maldonado (Madre de Dios – Peru), which was not included in previous morphological and genetic studies.

In addition to the genetic and morphological differences and the restricted geographic distributions (except for some regions of overlapping distributions), there is evidence for dissimilarities of chemical compounds and sexual behavior supporting the existence of independent biological entities in what has been referred to as *A. fraterculus* (Hendrichs et al., 2015). Studies of mating behavior have shown moderate to high‐level incompatibilities between some morphotypes (Juárez et al., 2015). This is the case for the Peruvian (lowlands of Peru) and the Brazilian‐1 (Argentina) morphotypes, which have shown assortative mating and displayed different pheromone chemical profiles that may promote pre‐zygotic isolation (Cáceres et al., 2009; Segura et al., 2011). The Mexican and Andean morphotypes also showed substantial sexual incompatibility with each other and the Brazilian‐1 and Brazilian‐3 morphotypes (Devescovi et al., 2014; Rull et al., 2013). Even though the differences among the Brazilian morphotypes seems more subtle, populations classified as Brazilian‐1 and Brazilian‐3 also showed different pheromone makeups (Vaníčková, Hernández‐Ortiz, et al., 2015), which is compatible with a certain degree of independent evolution.

The existence of phylogenetic lineages within the *A. fraterculus* complex implies that the effectiveness of species‐specific pest control strategies will depend on an accurate and rapid species diagnosis. Adequately identifying different lineages in the *fraterculus* group, and especially in the *A. fraterculus* complex, is particularly challenging due to ILS and introgression (Liu et al., 2017; Lopez‐Vaamonde et al., 2021). In such cases, multi‐locus DNA barcoding has been shown to be effective in species identification even with some levels of interspecific gene flow (Liu et al., 2017). A multi‐locus approach based on a panel of SNPs with high difference in allele frequencies (>0.95) has been developed to differentiate a pair of closely related tephritid species of the genus *Rhagoletis* (Doellman et al., 2020). The methods presented here can be applied to other taxa without necessarily having population‐scale data, and the only requirements would be a set of specimens with taxonomic identification and large‐scale genetic data (transcriptomes, genome assemblies and/or WGRs). Additionally, our approach based on clusters of orthologous genes is not limited to downstream applications using individual SNPs, but rather including entire multigene datasets. In this way, our methodology may be employed to generate diagnostic approaches for other arthropod pests, especially because of the abundant number of cryptic species complexes (Ashfaq & Hebert, 2016). Furthermore, its application is not limited to arthropods, but to any group of species where classical DNA barcodes have failed to accurately discriminate species. The extensive literature on this matter includes examples of diverse group of animals, such as the Blacktip complex (sharks), *Hippocampus kuda* complex (seahorses), *Pelophylax ridibundus* complex (water frogs), *Amazona aestiva*/*A. ochrocephala* species complex (parrots) (Cardeñosa et al., 2020; Gonçalves et al., 2015; Hawlitschek et al., 2016; Serite et al., 2021), as well as plants such as the *Solanum* section *Petota* (wild potatoes) and species of the genera *Salix* (trees) and *Veronica* (speedwell) (Spooner, 2009; Wyler & Naciri, 2016).

Our findings indicate that, despite the complex evolutionary history of the *fraterculus* group, it is possible to recover well‐supported clades, most of which have been associated with already described species, using a set of highly informative loci. We also showed that most of those loci (97) are located sparsely across the genome and may be considered as independently inherited. Notably, a subsampling analysis of those genes suggests that subsets of 80 loci provide clade formation concordant with the phylogeny generated for a set of more than 3000 loci, and almost as much phylogenetic resolution as was recovered by randomly selected sets of 700 loci. Nevertheless, two clades (Clade 2 and Clade 5) showed high‐topological conflict and were particularly insensitive to the increase in number of loci (Figure 6; Figure S4). If introgression is the main source of these incongruences, we would expect to have recovered those clades with reticulation in the networks. As the networks did not show reticulations directly associated with Clade 2 and Clade 5, we suggest that the informative set of genes may be recalcitrant to the impacts of gene flow (Congrains et al., 2021), even for *A. fraterculus* lineages. However, this result does not allow us to totally rule out introgression as the cause of the incongruence of gene trees produced by informative loci. Our assessment also shows that a smaller set of ~30 markers could be highly efficient at identifying different lineages in the *fraterculus* group and even provide phylogenetic information, except for a handful of cases. Due to the limited sampling, these findings should be treated with caution, but they undoubtedly form a cornerstone to develop tools for biosurveillance using multigene phylogenetics. Proper species identification in the genus *Anastrepha* has been a complex endeavor normally relegated to expert taxonomists, especially identifications in the *fraterculus* group. And even for these specialists, identification of males and early life stages, which are commonly detected by regulatory agencies, can be difficult to impossible. Therefore, our results are relevant to the use of molecular markers to aid in these identification tasks, thus democratizing some aspects of *Anastrepha* identification.

## 5 CONCLUSIONS

This study exemplifies how knowledge of the evolutionary patterns at the genomic scale coupled with modern phylogenomic approaches can be applied to discriminate lineages in the gray zone of speciation (de Queiroz, 2007). For that, we used methodology to integrate different sources of ‐omics data to produce accurate phylogenies, which was applied to assess the intricate evolutionary history of the *A. fraterculus* complex and related species. Our findings show that subsampling and gene tree discordance analysis are invaluable tools to determine the minimum number of loci needed to reasonably resolve a phylogeny and to recover particularly problematic clades. We found that only 80 genes provide similar phylogenetic resolution as the 3031 loci of the complete data set, and a set of only 30 markers may potentially be used for species discrimination of these important pests. This finding offers the foundation for a multi‐locus approach that could be used for regulatory agencies to efficiently monitor borders or establish quarantines. Additionally, as sequencing techniques become cheaper and more flexible, future studies should focus on developing cost‐effective methods to test the efficiency of these loci in other populations and closely related species.

## CONFLICT OF INTEREST STATEMENT

The authors declare that there is no conflict of interest.

## Supporting information


Data S1.

## Data Availability

Whole‐genome resequencing data obtained for this study has been deposited to the Sequence Read Archive (SRA) of the GenBank under the accession numbers (SRR22490025‐SRR22490037).
