# Supplementary material for: Phylogenomic analysis provides diagnostic tools for the identification of Anastrepha fraterculus (Diptera: Tephritidae) species complex
Source: Evol Appl. 2023 Aug 30;16(9):1598–618. doi: 10.1111/eva.13589 (PMC10519418; doi:10.1111/eva.13589)
Supplement: Supplementary file 1 — Data S1. [file EVA-16-1598-s001.docx]

**Supplementary Material**

**Table S1.** Number of raw and filtered pair-end reads of whole genomes sequencing of *Anastrepha*.

| Sample | Sequenced PE reads | Read length (bp) | Filtered PE reads |
| --- | --- | --- | --- |
| *A. fraterculus* RS2 | 53,861,567 | 150 | 49,600,417 |
| *A. fraterculus* MX | 41,472,177 | 150 | 39,197,991 |
| *A. fraterculus* CUS | 78,391,785 | 150 | 73,564,362 |
| *A. fraterculus* LOR | 68,626,728 | 150 | 64,192,841 |
| *A. fraterculus* ANC | 63,893,156 | 150 | 59,990,432 |
| *A. fraterculus* MDD | 93,085,825 | 150 | 87,312,536 |
| *A. fraterculus* CO | 56,356,316 | 150 | 52,999,725 |
| *A. fraterculus* EC | 87,883,104 | 150 | 81,981,094 |
| *A. striata* | 97,675,963 | 150 | 91,555,494 |
| *A. hadracantha* | 87,008,565 | 150 | 82,740,820 |
| *A. leptozona* | 53,141,838 | 150 | 50,408,633 |
| *A. curitis* | 77,644,243 | 150 | 72,567,226 |
| *A. psidivora* | 80,547,456 | 150 | 74,642,047 |


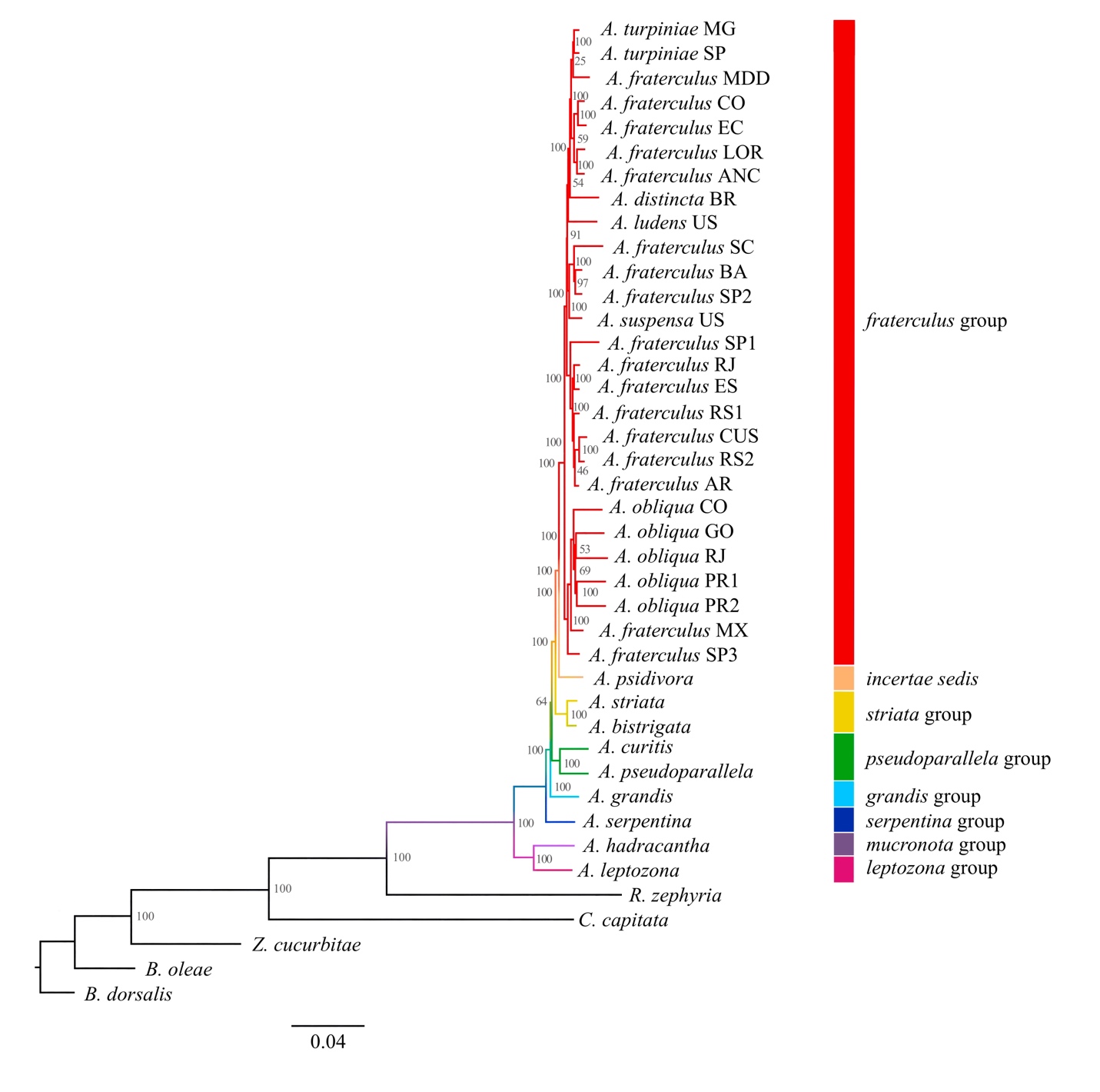


**Figure S1**. Concatenated phylogeny of *Anastrepha* and five other Tephritidae species as outgroups based on 2,591 genes inferred in IQ-TREE. Bootstrap supports are shown close to the nodes.


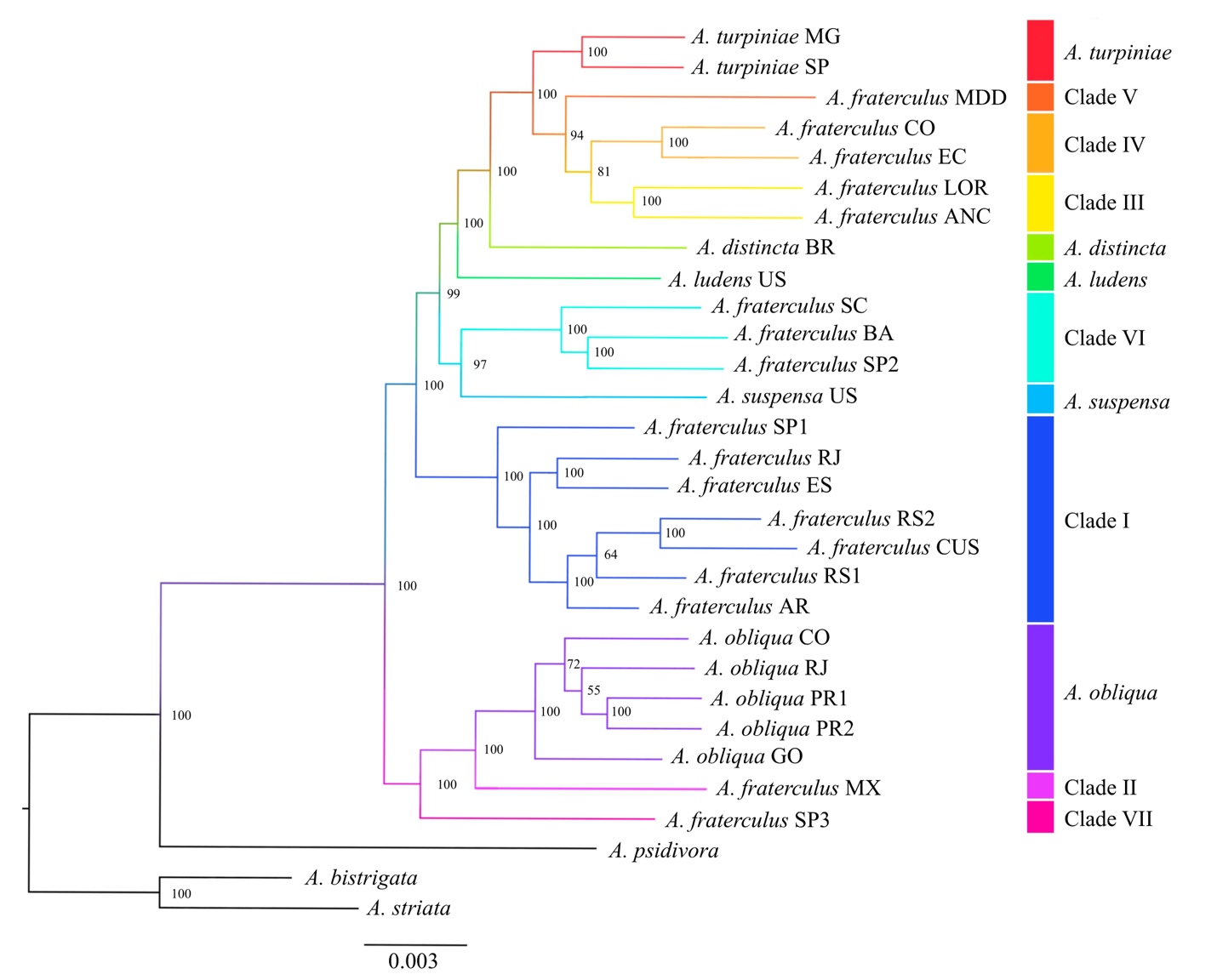


**Figure S2**. Concatenated phylogeny of *fraterculus* group based on 3,031 genes using *A. psidivora*, *A. bistrigata* and *A. striata* as outgroups. Bootstrap supports are shown close to the nodes.


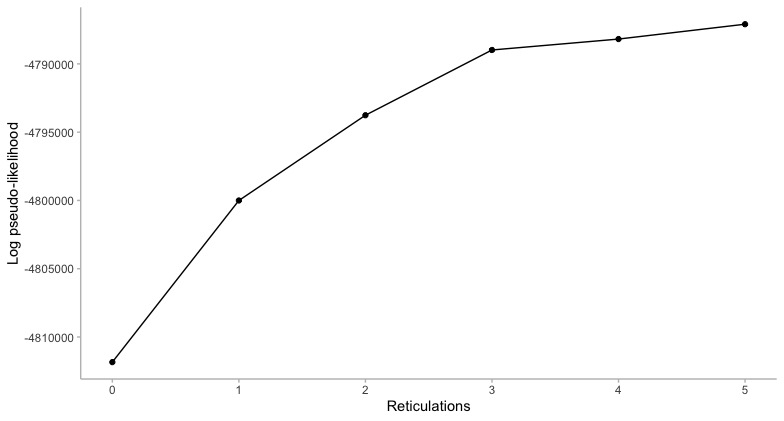


**Figure S3**. Log pseudo-likelihood of networks inferred based on 3,031 genes of *A. fraterculus* group lineages. Stationary phase of log pseudo-likelihood starts at three reticulations.


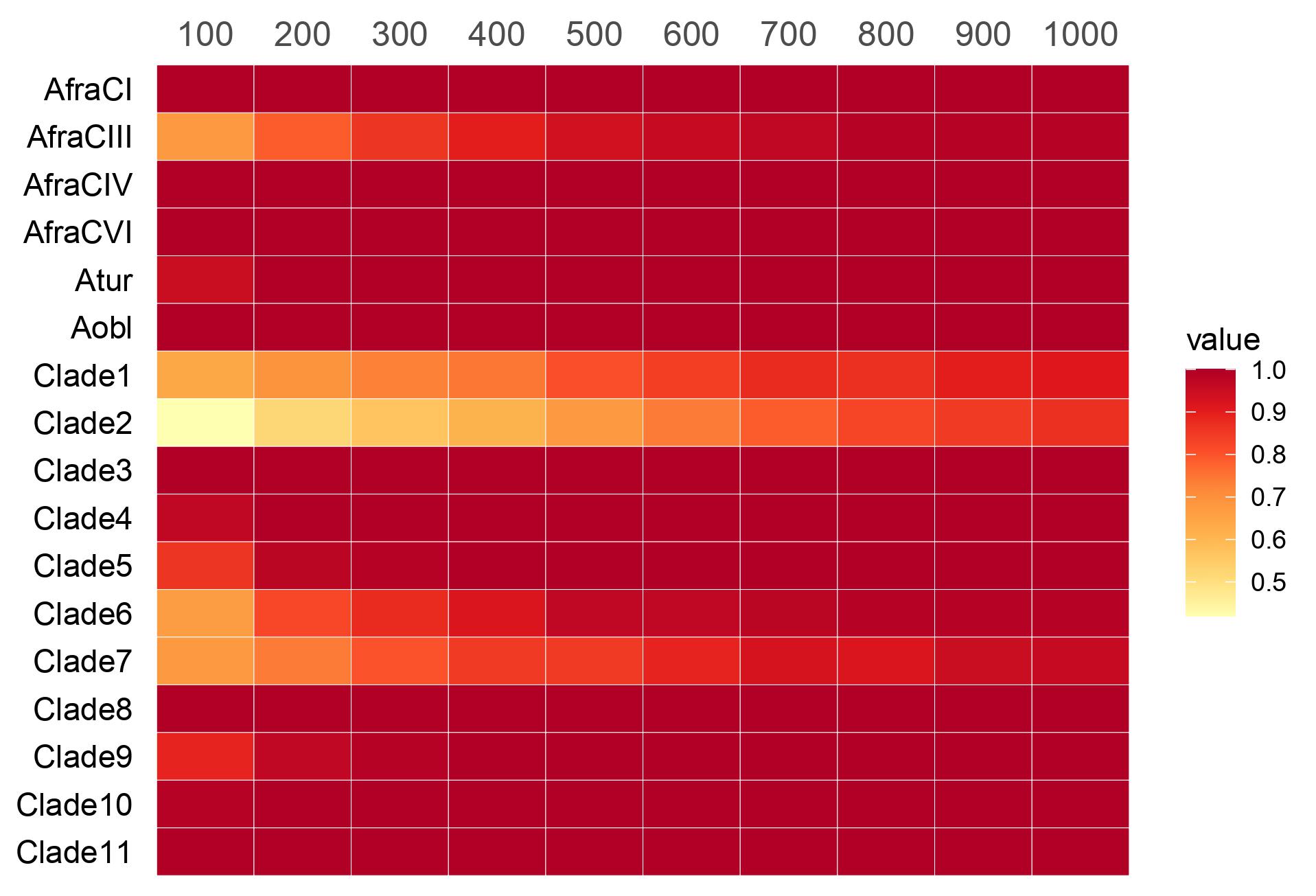


**Figure S4**. Phylogenetic congruence analysis of gene tree subsamples derived from the whole dataset. Heatmaps of the proportion of clades recovered for each class containing subsets of increasing number of gene trees (100, 200, 300, 400, 500, 600, 700, 800, 900, 1000) randomly sampled from the whole dataset. Each category included the information of 500 species trees inferred from independently subsampled set of gene trees. The clades are labeled in Figure 6A.


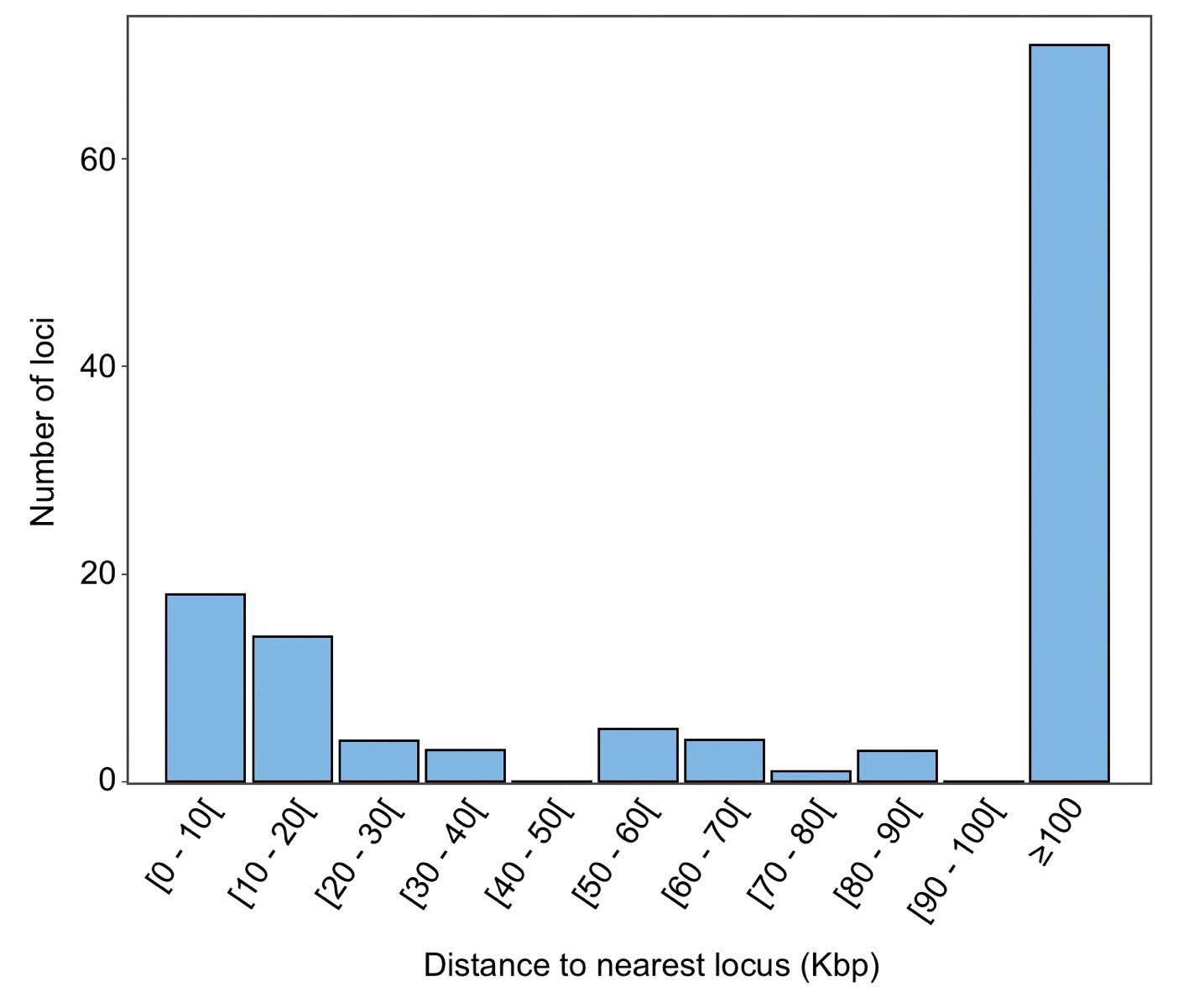


**Figure S5**. Distribution of the distance to the closest locus. The distances were calculated between loci based on the location of the phylogenetically informative set (123 genes) in the *A. ludens* genome.
